# The Lower Extremity of the Ulna as a Factor in Colles’s Fracture1Read at the Thirty-eighth Annual Meeting of the Medical Association of Central New York, held at Buffalo, October, 24, 1905.

**Published:** 1906-07

**Authors:** D. M. Totman

**Affiliations:** Syracuse, N. Y.


### The Lower Extremity of the Ulna as a Factor In Colles's Fracture.[1]

By D. M. TOTMAN' M. D., Syracuse, N. Y.

IT is now thirty-five years since Dr. Moore presented his first paper before the Medical Society of the State of New York, upon "A Luxation of the Ulna not heretofore described." In 1880, just ten years after the first paper, Dr. Moore reaffirmed his first views—namely, that there was a true luxation of the lower end of the ulna; that this was accompanied by a fracture of the styloid process of the ulna, which fractured portion remained attached to the external lateral ligament; that this fracture left a peculiar wedge-shaped part on the ulna, which could and did get entangled in the annular ligament which winds around the wrist at this point. My own experience confirms me in this regard. This entanglement does occur, and if it is not corrected a deformity will

1. Read at the Thirty-eighth Annual Meeting of the Medical Association of Central New York, held at Buffalo, October, 24, 1905.

surely result. It will never free itself, that is certain, and if left in this position it will become fixed.

Dr. Moore also taught that the radio-ulnar articulation was generally torn and the synovial membrane in this locality generally ruptured. This synovial membrane rests upon the triangular fibrocartilage. It takes its origin from the rim and side of the radius and, covering the head of the ulna, is inserted into the pit at the root of the styloid process.

The claim was made that Dr. Moore reported dissections of extreme cases; and so they were out of the line of a regular Colles's fracture, and as such, not met with in everyday practice. However, Dr. Moore's findings have been fully confirmed I think, practically, in every particular. The *x*-rays certainly have confirmed his contentions of the fracture of the styloid process, as it is indeed rare to see an *x*-ray of a Colles's fracture in which this is not clearly shown. And I want to bear tribute to this wonderfully skilful worker and investigator in this particular field. The profession is, however, tardy in accepting the teachings for which he so long contended, not for his own fame, but for the advancement of the great profession which he so nobly adorned. I am sure you will pardon me for speaking thus feelingly on this subject.

The subject matter which I bring before you first had its origin in a case in which there was a bad deformity, and an almost useless hand. It had appeared to me that there was nonunion in the radial fracture, and that the lower end of the radial shaft pressed well down into the anterior wrist under the flexor tendons, where is was pressing on the median nerve. There also appeared to be an apparent lengthening and enlargement of the lower end of the ulna. I consulted with Dr. Moore about this case and though he did not see the patient, he yet told me that he thought I was mistaken about the nonunion, as he had never seen a nonunion of the lower end of the radius which he furthermore told me was the best bone in the body to unite. He had always found a fracture here united in some way.

I subsequently operated on this case and found there was nonunion; then the fragments were brought in apposition and wired. After they were so brought together I found the radius was much shorter than the ulna. The hand was still crowded around to the radial side. Seeing no other way to correct this condition, I exsected half an inch of the lower end of the ulna. This was greatly enlarged by what appeared to me to be new and excessive bone deposit at the lower end of the ulna. After the exsection I found the surrounding parts covered with a bony exudate which felt like coarse sand paper. I curetted this most thoroughly. The parts healed well and I congratulated myself in getting a fairly good result. I was destined however to severe disappointment. There was a rapid filling in of bone in the place where I had exsected the ulna, and in the end it was larger than before the exsection. The hand was crowded more than before to the radial side and I was sure there was atrophy of the radius. The radius united with bony union but it was very tardy and slow.

My second observation followed soon after this case. A man was brought into St. Joseph's Hospital, Syracuse, with severe head and back injuries, and with what appeared to be Colles's fracture of the left wrist. He was unconscious and so could tell nothing about it. After examination it was found the wrist injury was an old affair. The man subsequently died and the autopsy gave me the specimen which I show you, with two well marked bony formations,—one at the site of the styloid process and the other at the radioulnar articulation. In this case there was the well marked prominence of the ulna and the hand was crowded to the radial side.

My third case, which I wish to relate, was a patient under my own care. He was admitted to the hospital with a fracture of the left thigh, about the middle, and some crushing injury of the left pelvis. He also had what appeared to be a typical Colles's fracture of the left wrist. The patient was delirious at times and succeeded in pulling all the dressing from the wrist and the deformity recurred. It was again replaced and the hand was put up in a plaster splint. At the end of the fourth week there was an undue prominence of the ulna with deformity, the hand being crowded to the radial side.

The patient was anesthetized and an attempt was made to reduce the parts for the third time; this time the hand was dressed as in the first reduction, using the Moore dressing, with this addition—namely, that the hand was placed in a Levis's splint. The final result is shown by this *x*-ray, which was taken two years after the injury. There is an ugly deformity, the hand being crowded to the radial side. The ulna is prominent laterally, and appears as if the radius was shortened, also as if the bone was smaller than the right. The flexion and extension of the hand is very much limited and the patient says it is still painful at times. The case in appearance is one which could easily figure in a malpractice suit. Now I could present other and similar cases to these but I think they are familar to you all.

My contention is this: that there are cases occasionally met with in which the injuries of the lower end of the ulna are such that bony formations will occur from the injury to the periosteum and the other adjacent structures. We now know that the styloid process is almost always torn off, which certainly must involve the periosteum at this point.

This recalls to my mind an interesting episode in one of my patients. Col. T. Suffered an excision of his right elbow from a gunshot wound in the civil war. While walking to his stable he was thrown by his spaniel running between his legs. He suffered a typical Colles's fracture as shown by an *x*-ray. The *x*-ray was not taken by a medical man, but he told my patient that there was a bone loose in his wrist. I was immediately sent for in great haste and I found the patient greatly excited. I was told that there was a bone loose, and was asked what I was going to do about it. I had no little trouble in quieting the fears of my patient.

In conclusion: the most important fact which is to be deduced is that in this luxation of the ulna, serious injuries are sustained in its lower end, and that in certain cases deformity will result in spite of the very best care; that such cases are quite liable to subject the surgeon to a malpractice suit; that if these facts are true they should be well established in the working resources of the profession.

I have still to call attention to one other point, and that is the tearing loose and displacement of the tendon of the extensor carpiulnaris where it passes in its groove over the lower end of the ulna. If I have read Dr. Moore's teachings aright, and my studies of this fracture cover nearly thirty years of work, with three careful dissections of this injury, I wish to reaffirm that the care of the lower end of the ulna is the key to the successful treatment of Colles's fracture. If the luxation is successfully reduced, and if the ulna is held up in its proper normal place, it makes the best possible splint for the fractured radius. This can best be accomplished by the use of a small roller under the outer and anterior surface, and this is held in place with a firm encircling adhesive plaster. If bony exostosis does occur it will be reduced to a minimum, and a suit for malpractice can be successfully defended.

In all of this I would not leave the impression that I ignore the fractured radius. That could well make another paper. I must say, however, that I never stop in my reduction of a Colles's fracture, until all the parts are as near right as I can possibly make them, and the sine qua non in reference to this is the obtaining of the normal concavity at the lower and anterior surfaces of the radius.

393 Montgomery Street.

